# Supplementary material for: Identification of photoperiod-induced specific miRNAs in the adrenal glands of Sunite sheep (Ovis aries)
Source: Front Vet Sci. 2022 Jul 22;9:888207. doi: 10.3389/fvets.2022.888207 (PMC9354845; doi:10.3389/fvets.2022.888207)
Supplement: Supplementary file 1 [file Data_Sheet_1.ZIP › Supplementary file/miRDeep2 file.zip!/miRDeep2 file/Novel_Structure/LP42A2/Novel_694.pdf]

Provisional ID	:	NC_019463.2_14502
Score total	:	6.3
Score for star read(s)	:	3.9
Score for read counts	:	-1.3
Score for mfe	:	2.2
Score for randfold	:	1.6
Score for cons. seed	:	
Total read count	:	9
Mature read count	:	2
Loop read count	:	0
Star read count	:	7

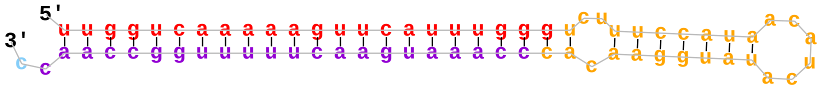

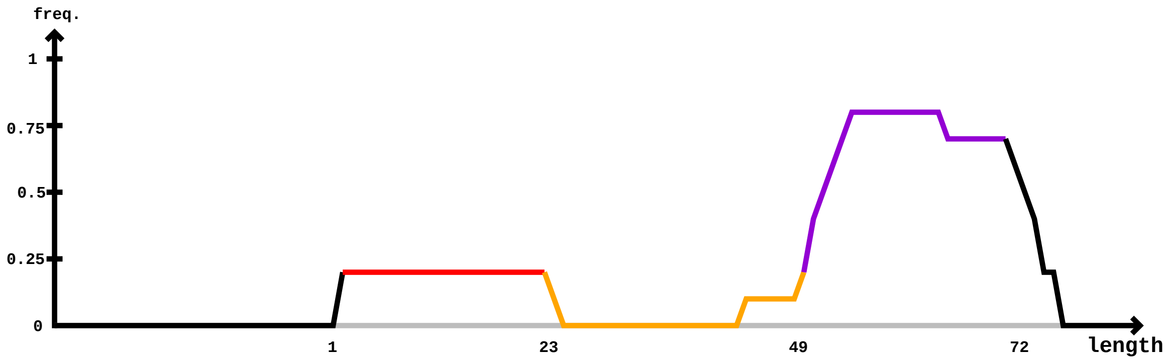

Mature **Star**

```
5'- aaagcauucuuuuauguccacuaauagguuggucaaaaaguucauuugggucuuuccauaacaucauauggaacacccaaaugaacuuuuuggccaacccauacaucauac   -3'    obs
    aaagcauucuuuuauguccacuaauagguuggucaaaaaguucauuugggucuuuccauaacaucauauggaacacccaaaugaacuuuuuggccaacccauacaucauac          exp
    ...((((......)))).........(((((((((((((((((((((((((..(((((((......))))))).))))))))))))))))))))))))).............   reads    mm    sample
    ............................uuggGcaaaaaguucauuuggg.............................................................   2        1     seq
    ......................................................................gaacacccaaaugaacuuuuu....................   1        0     seq
    ............................................................................ccaaaugaacuuuCuggccaac.............   1        1     seq
    .............................................................................Uaaaugaacuuuuuggccaac.............   1        1     seq
    .............................................................................caaaugaacuuuuuggccaacA............   1        1     seq
    ..............................................................................aaaugaacuuuuuggccaaccca..........   1        0     seq
    ...............................................................................aaugaacuuuuuggccaaccca..........   1        0     seq
    ................................................................................augaacuuuuuggccaacccaGa........   1        1     seq
    .................................................................................ugaacuuuuuggccaacccaua........   1        0     seq
```
